# The impact of second transurethral resection on survival outcomes in patients with non-muscle-invasive bladder cancer treated with bacillus Calmette–Guérin therapy

**DOI:** 10.1093/jjco/hyad155

**Published:** 2023-11-16

**Authors:** Hiroshi Kikuchi, Takashige Abe, Makito Miyake, Haruka Miyata, Ryuji Matsumoto, Takahiro Osawa, Nobutaka Nishimura, Kiyohide Fujimoto, Junichi Inokuchi, Takahiro Yoneyama, Ryotaro Tomida, Kazuyuki Numakura, Yuto Matsushita, Kazumasa Matsumoto, Takuma Sato, Rikiya Taoka, Takashi Kobayashi, Takahiro Kojima, Yoshiyuki Matsui, Naotaka Nishiyama, Hiroshi Kitamura, Hiroyuki Nishiyama, Nobuo Shinohara

**Affiliations:** Department of Urology, Hokkaido University Graduate School of Medicine, Sapporo, Japan; Department of Urology, Hokkaido University Graduate School of Medicine, Sapporo, Japan; Department of Urology, Nara Medical University, Kashihara, Nara, Japan; Department of Urology, Hokkaido University Graduate School of Medicine, Sapporo, Japan; Department of Urology, Hokkaido University Graduate School of Medicine, Sapporo, Japan; Department of Urology, Hokkaido University Graduate School of Medicine, Sapporo, Japan; Department of Urology, Nara Medical University, Kashihara, Nara, Japan; Department of Urology, Nara Medical University, Kashihara, Nara, Japan; Department of Urology, Graduate School of Medical Sciences, Kyushu University, Fukuoka, Japan; Department of Urology, Hirosaki University Graduate School of Medicine, Hirosaki, Japan; Department of Urology, National Hospital Organization Shikoku Cancer Center, Matsuyama, Ehime, Japan; Department of Urology, Akita University Graduate School of Medicine, Akita, Japan; Department of Urology, Hamamatsu University School of Medicine, Hamamatsu, Japan; Department of Urology, Kitasato University School of Medicine, Sagamihara, Japan; Department of Urology, Tohoku University Graduate School of Medicine, Sendai, Japan; Departments of Urology, Kagawa University Faculty of Medicine, Takamatsu, Japan; Department of Urology, Kyoto University Graduate School of Medicine, Kyoto, Japan; Department of Urology, Aichi Cancer Center, Nagoya City, Aichi, Japan; Department of Urology, National Cancer Center Hospital, Tokyo, Japan; Department of Urology, Faculty of Medicine, University of Toyama, Toyama, Japan; Department of Urology, Faculty of Medicine, University of Toyama, Toyama, Japan; Department of Urology, Faculty of Medicine, University of Tsukuba, Tsukuba, Ibaraki, Japan; Department of Urology, Hokkaido University Graduate School of Medicine, Sapporo, Japan

**Keywords:** bacillus Calmette–Guérin, non-muscle invasive bladder cancer, second transurethral resection

## Abstract

**Objective:**

Several guidelines recommended that second transurethral resection should be performed in patients with diagnosis of high-risk non-muscle-invasive bladder cancer. However, therapeutic benefits of second transurethral resection before bacillus Calmette–Guérin intravesical instillation were conflicting amongst previous studies. We investigated the prognostic impact of second transurethral resection before bacillus Calmette–Guérin instillation in high-risk non-muscle-invasive bladder cancer patients.

**Methods:**

This retrospective study included 3104 non-muscle-invasive bladder cancer patients who received bacillus Calmette–Guérin instillations between 2000 and 2019 at 31 collaborative institutions. Univariate and multivariate Cox proportional hazards models were used to assess the risk factors of intravesical recurrence, disease progression, cancer-specific mortality and overall mortality.

**Results:**

In the entire population, patients undergoing second transurethral resection (33%, 1026/3104) had a lower risk of intravesical recurrence on univariate analysis (hazard ratio 0.85, 95% confidence interval 0.73–0.98, *P* = 0.027), although it did not remain significant on multivariate analysis (hazard ratio 0.90, 95% confidence interval 0.76–1.07, *P* = 0.24). Subgroup analysis revealed that, in pT1 patients (*n* = 1487), second transurethral resection was significantly correlated with a lower risk of intravesical recurrence on multivariate analysis (hazard ratio 0.80, 95% confidence interval 0.64–1.00, *P* = 0.048), but lower risks of disease progression (hazard ratio 0.75, 95% confidence interval 0.56–1.00, *P* = 0.049), cancer-specific mortality (hazard ratio 0.54, 95% confidence interval 0.35–0.85, *P* = 0.007) and overall mortality (hazard ratio 0.73, 95% confidence interval 0.55–0.97, *P* = 0.027) on univariate analysis.

**Conclusions:**

Second transurethral resection confers accurate pathological staging and could be used to safely select good candidates for intravesical bacillus Calmette–Guérin instillation. We further confirm that second transurethral resection could confer an oncological benefit in pT1 bladder cancer patients treated by bacillus Calmette–Guérin instillation, and so strongly recommend second transurethral resection in this patient population.

## Teaser

Second transurethral resection reduced intravesical recurrence, disease progression and cancer-specific mortality in pT1 bladder cancer patients treated by bacillus Calmette–Guérin instillation from the largest Japanese cohort.

## Introduction

In the management of patients with non-muscle-invasive bladder cancer (NMIBC), transurethral resection of a bladder tumour (TURBT) is an initial treatment step to eliminate all macroscopic diseases and obtain pathological information. Furthermore, in high-risk NMIBC patients including high-grade (HG) or grade 3, Ta or T1 cancers (1,2), in order to avoid pathological under-staging, resect residual cancer, and correctly identify, for example, which patient needs immediate radical cystectomy or could be a candidate for bladder preservation therapy (3), several major guidelines recommend second transurethral resection (TUR).

In high-risk NMIBC, in order to reduce cancer recurrence and the progression risk, bacillus Calmette–Guérin (BCG) has been widely utilized. Therapeutic benefits of second TUR before BCG intravesical instillation have been conflicting amongst studies. Iida et al. (4) observed that patients with second TUR showed significantly better intravesical recurrence-free survival (RFS) than those without second TUR after BCG therapy, whereas Baba et al. (5) did not. In a large multi-institutional retrospective cohort of 2451 patients with BCG-treated HG T1 tumours (second TUR was performed in 935 patients), second TUR improved treatment outcomes when muscle was not present in the initial TUR specimen (6). In our hospital (Hokkaido University hospital), we actively carried out second TUR for high-risk NMIBC patients before BCG instillation, and observed residual tumours in 49.5% (54/109) at the second TUR, and good caner control outcomes [2-year RFS of 80.7%, and 2-year progression-free survival (PFS) of 85.7%] after BCG instillation (7).

Recently, the Japanese Urological Oncology Group (JUOG) retrospectively collected large cohort data (>3000) on NMIBC treated by BCG instillation, and reported several studies, such as non-maintenance schedule outcomes (8), or poorer disease control in an older population (≥ 75 years) (9). Using this large JUOG cohort, in order to gain further insight into the treatment role of second TUR before BCG instillation, we evaluated the survival impact of second TUR before BCG therapy in high-risk NMIBC patients.

## Patients and methods

### Study population

This multicentre retrospective study was approved by the institutional review board of each participating institute (reference protocol ID: 2217) of the JUOG framework. We reviewed 3280 patients who received TURBT followed by intravesical BCG treatment between 2000 and 2019 at 31 collaborative institutions. After excluding patients with muscle-invasive disease (≥ pT2) or unknown pT stage (*n* = 45), and those with unknown treatment outcomes (*n* = 119), 3104 NMIBC patients were included in the present study. Patient characteristics, including age, sex, tumour status (primary or recurrent), tumour number, tumour size, pathology, second TUR, BCG treatment schedule, BCG strains and treatment outcomes, were retrospectively collected. The pathological diagnosis was determined by pathologists at each institution, not by a central pathologist. For the analysis, the tumour grade was stratified according to the 2004 WHO classification.

### Treatment and follow-up protocol

Second TUR was performed based on physicians’ discretion. The BCG schedule was weekly instillation of Immunobladder^®^ (Tokyo-172 strain) or ImmuCyst^®^ (Connaught strain) for 6–8 consecutive weeks for induction BCG with or without subsequent maintenance BCG. Regarding the maintenance BCG schedule, in general, it was once a week for 3 weeks at 3, 6, 12, 18, 24, 30 and 36 months after the first induction BCG. After BCG therapy, patients were followed up with cystoscopy and urinary cytology every 3 months for 2 years, then every 6 months in the 3rd and 4th years, and then annually thereafter.

### Statistical analysis

Clinicopathological characteristics were compared using chi-square and Mann–Whitney *U* tests, as appropriate. Survival estimates were analyzed using the Kaplan–Meier method and compared using the log-rank test. RFS, PFS, cancer-specific survival (CSS) and overall survival (OS) were calculated from the date of the first BCG to that of each first event or last visit. In the present study, intravesical recurrence was defined as recurrent tumours of pathologically proven urothelial carcinoma in the bladder and prostatic urethra. Disease progression was defined as recurrent disease with muscle invasion (pT2), development of regional lymph node swelling and/or distant metastases or bladder cancer death. Death other than from bladder cancer was censored in PFS and CSS analyses. Cox proportional hazards models were used to analyze the associations between the clinical characteristics, including age, sex, tumour status (primary/recurrent), pathology, tumour multiplicity, tumour size, variation of BCG instillation (schedule, strain) and second TUR, with RFS, PFS, CSS and OS. We first performed these survival analyses in the total cohort and then performed sub-analyses in pT1 and pTa cohorts. JMP version 16 (SAS Institute, Japan) was used for all calculations, and *P* < 0.05 was considered to be significant.

## Results

The clinical and pathological features of the present cohort divided by a second TUR performance (Yes vs. No) are shown in Table 1. Overall, 33% of total patients received second TUR. In pTa disease patients, 16.7% (166/995) underwent second TUR, and 56.3% (837/1487) did in the pT1 disease cohort. Nine factors were significantly different between patients with and without second TUR. For example, patients who received second TUR were younger than those who did not (70 vs. 72 years, respectively, *P* < 0.0001), and had higher proportions of HG tumours (*P* < 0.0001), pT1 disease (*P* < 0.0001) and large tumours (*P* < 0.0001). On pathological analyses at the second TUR, residual tumours were detected in 53.6% of pTa patients (89/166) and 51% of pT1 patients (430/837) (Supplementary Table S1).

**Table 1:** Baseline characteristics of patients for entire cohort

	**Second TUR**		***P*-value**
**Variables**	**Yes**	**No**	
	***n* = 1026**	***n* = 2078**	
Age, year, median (interquartile range)	70 (64–77)	72 (66–78)	**<0.0001**
Sex, no. (%)			0.069
Male	825 (80.4)	1726 (83.1)	
Female	201 (19.6)	352 (16.9)	
Primary or recurrent, *n* (%)			**<0.0001**
Primary	936 (91.2)	1388 (66.8)	
Recurrent	90 (8.8)	689 (33.1)	
Unknown	0 (0.0)	1 (0.1)	
Pathological tumour grade, no. (%)			**<0.0001**
Low	25 (2.4)	280 (13.5)	
High	994 (96.9)	1696 (81.6)	
Unknown	7 (0.7)	102 (4.9)	
pT stage, no. (%)			**<0.0001**
pTa	166 (16.2)	829 (39.9)	
pT1	837 (81.6)	650 (31.3)	
Primary CIS	23 (2.2)	599 (28.8)	
Concurrent CIS^a^, no. (%)			**0.0004**
Yes	276 (27.5)	507 (34.3)	
No	726 (72.4)	965 (65.2)	
Unknown	1 (0.1)	7 (0.5)	
Tumour multiplicity, no. (%)			**<0.0001**
Single	362 (35.3)	5532 (26.6)	
Multiple	646 (62.9)	1457 (70.1)	
Unknown	18 (1.8)	69 (3.3)	
Tumour size, no. (%)			**<0.0001**
<3 cm	627 (61.1)	1238 (59.6)	
≥3 cm	271 (26.4)	226 (10.9)	
Unknown	128 (12.5)	614 (29.5)	
Maintenance BCG, no. (%)			0.58
Yes	172 (16.8)	365 (17.6)	
No	854 (83.2)	1713 (82.4)	
No. BCG instillations at induction			**<0.0001**
≥9	1 (0.1)	4 (0.2)	
7/8	530 (51.6)	892 (42.9)	
5/6	442 (43.1)	1109 (53.4)	
≤4	50 (4.9)	68 (3.3)	
Unknown	3 (0.3)	5 (0.2)	
BCG strain used, *n* (%)			**0.0045**
Tokyo–172	821 (80.0)	1555 (74.8)	
Connaught	203 (19.8)	514 (24.8)	
Unknown	2 (0.2)	9 (0.4)	
Follow-up, months, median (interquartile range)	49 (26–78)	48 (26–72)	**0.0050**

^a^Concurrent CIS rates were counted in Ta/T1 cases. TUR, transurethral resection; BCG, bacillus Calmette–Guérin.

Table 2 summarizes uni- and multivariate analyses of intravesical recurrence, disease progression, cancer-specific mortality and overall mortality in the entire cohort. Older age [hazard ratio (HR) 1.02, 95% confidence interval (CI) 1.01–1.03, *P* < 0.0001), recurrent tumour (HR 1.36, 95% CI 1.13–1.65, *P* = 0.001), multiple tumours (HR 1.34, 95% CI 1.13–1.59, *P* < 0.001) and ≥ 3 cm tumour (HR 1.50, 95% CI 1.24–1.81, *P* < 0.0001) were independently significant adverse factors, and maintenance BCG instillation reduced intravesical recurrence (HR 0.43, 95% CI 0.34–0.56, *P* < 0.0001) in the multivariate model. Second TUR did not remain significant in the multivariate model, although it was significant in the univariate model (HR 0.85, 95% CI 0.73–0.98, *P* = 0.027). In terms of disease progression, older age (HR 1.03, 95% CI 1.02–1.05, *P* < 0.001) and pT1 disease (HR 2.58, 95% CI 1.70–3.92, *P* < 0.0001) were independently significant adverse factors, and maintenance BCG reduced disease progression (HR 0.55, 95% CI 0.35–0.88, *P* = 0.012) in the multivariate model. Regarding CSS, older age (HR 1.07, 95% CI 1.05–1.10, *P* < 0.0001), recurrent tumour (HR 2.01, 95% CI 1.29–3.12, *P* = 0.002) and pT1 disease (HR 3.17, 95% CI 1.94–5.20, *P* < 0.0001) were significantly adverse risk factors. An older age (HR 1.08, 95% CI 1.06–1.10, *P* < 0.0001) and pT1 disease (HR 1.44, 95% CI 1.08–1.92, *P* = 0.014) were significant adverse risk factors for OS. Females had better OS than males (HR 0.63, 95% CI 0.44–0.90, *P* = 0.019).

**Table 2:** Univariate and multivariate Cox proportional hazards model predicting intravesical recurrence, disease progression, cancer-specific mortality and overall mortality

	*N*	**Intravesical recurrence**				**Disease progression**				**Cancer-specific mortality**				**Overall mortality**			
		Univariate		Multivariate		Univariate		Multivariate		Univariate		Multivariate		Univariate		Multivariate	
		HR (95% CI)	*P*-value	HR (95% CI)	*P*-value	HR (95% CI)	*P-*value	HR (95% CI)	*P-*value	HR (95% CI)	*P-*value	HR (95% CI)	*P-*value	HR (95% CI)	*P-*value	HR (95% CI)	*P-*value
Age (years) (Each increase of 1 year)		1.02 (1.01–1.03)	**<0.0001**	1.02 (1.01–1.03)	**<0.0001**	1.03 (1.02–1.05)	**<0.0001**	1.03 (1.02–1.05)	**<0.001**	1.07 (1.05–1.10)	**<0.0001**	1.07 (1.05–1.10)	**<0.0001**	1.08 (1.07–1.10)	**<0.0001**	1.08 (1.06–1.10)	**<0.0001**
Sex Male Female	2551 553	Ref. 1.00 (0.84–1.19)	0.97			Ref. 0.98 (0.73–1.32)	0.90			Ref. 0.78 (0.49–1.25)	0.30			Ref. 0.65 (0.48–0.89)	**0.007**	Ref. 0.63 (0.44–0.90)	**0.019**
Tumour status Primary Recurrent	2324 779	Ref. 1.45 (1.26–1.67)	**<0.0001**	Ref. 1.36 (1.13–1.65)	**0.001**	Ref. 1.07 (0.83–1.39)	0.59			Ref. 1.52 (1.07–2.17)	**0.019**	Ref. 2.01 (1.29–3.12)	**0.002**	Ref. 1.15 (0.91–1.45)	0.24		
Pathological tumour grade Low High	305 2690	Ref. 1.02 (0.81–1.29)	0.84			Ref. 1.91 (1.16–3.16)	**0.012**	Ref. 1.05 (0.53–2.11)	0.88	Ref. 1.64 (0.80–3.36)	0.17			Ref. 2.29 (1.37–3.85)	**0.002**	Ref. 1.74 (0.96–3.15)	0.066
pT stage Ta T1	995 1487	Ref. 1.08 (0.93–1.26)	0.31			Ref. 2.34 (1.73–3.17)	**<0.0001**	Ref. 2.58 (1.70–3.92)	**<0.0001**	Ref. 2.12 (1.35–3.31)	**0.001**	Ref. 3.17 (1.94–5.20)	**<0.0001**	Ref. 1.41 (1.09–1.81)	**0.008**	Ref. 1.44 (1.08–1.92)	**0.014**
Concurrent CIS No Yes	1691 783	Ref. 1.09 (0.93–1.28)	0.28			Ref. 1.24 (0.95–1.61)	0.11			Ref. 0.95 (0.63–1.43)	0.80			Ref. 1.14 (0.89–1.46)	0.30		
Tumour multiplicity Single Multiple	914 2103	Ref. 1.35 (1.16–1.58)	**<0.001**	Ref. 1.34 (1.13–1.59)	**<0.001**	Ref. 1.23 (0.95–1.59)	0.12			Ref. 1.51 (1.00–2.27)	**0.049**	Ref. 1.27 (0.83–1.95)	0.28	Ref. 1.14 (0.90–1.44)	0.29		
Tumour size <3 cm ≥3 cm	1865 497	Ref. 1.31 (1.09–1.57)	**0.003**	Ref. 1.50 (1.24–1.81)	**<0.0001**	Ref. 1.56 (1.15–2.11)	**0.004**	Ref. 1.33 (0.96–1.84)	0.089	Ref. 1.23 (0.78–1.93)	0.38			Ref. 0.95 (0.70–1.28)	0.72		
Maintenance BCG No Yes	2567 537	Ref. 0.44 (0.35–0.55)	**<0.0001**	Ref. 0.43 (0.34–0.56)	**<0.0001**	Ref. 0.58 (0.41–0.82)	**0.002**	Ref. 0.55 (0.35–0.88)	**0.012**	Ref. 0.69 (0.42–1.14)	0.15			Ref. 0.66 (0.48–0.90)	**0.010**	Ref. 0.81 (0.56–1.16)	0.24
No. BCG instillations at induction ≥6 <6	2911 184	Ref. 1.29 (0.99–1.68)	0.058			Ref. 1.13 (0.71–1.79)	0.62			Ref. 1.48 (0.80–2.75)	0.21			Ref. 1.54 (1.06–2.23)	**0.024**	Ref. 1.21 (0.78–1.88)	0.39
BCG strain used Tokyo–172 Connaught	2376 717	Ref. 1.14 (0.98–1.32)	0.087			Ref. 0.96 (0.74–1.24)	0.76			Ref. 0.86 (0.59–1.25)	0.43			Ref. 0.97 (0.77–1.20)	0.97		
Second TUR No Yes	1026 2078	Ref. 0.85 (0.73–0.98)	**0.027**	Ref. 0.90 (0.76–1.07)	0.24	Ref. 1.06 (0.84–1.35)	0.61			Ref. 0.76 (0.52–1.12)	0.16			Ref. 0.90 (0.71–1.13)	0.36		

HR, hazard ratio; CI, confidence interval.

**Figure 1:**
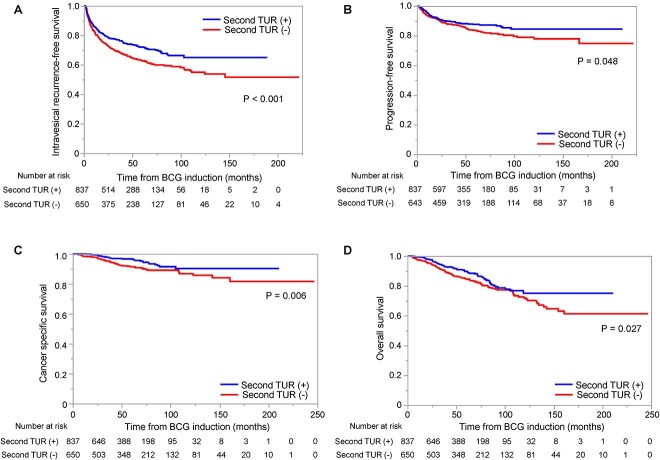
Kaplan–Meier survival analysis displaying (A) intravesical recurrence-free survival (RFS), (B) progression-free survival (PFS) (C) cancer-specific survival (CSS) and (D) overall survival (OS) in patients with T1 disease.

Figures 1 and S1 show sub-analyses of RFS, PFS, CSS and OS divided by the performance of second TUR and pT stage. Second TUR was associated with better RFS, PFS, CSS and OS (*P* < 0.001 and *P* = 0.048, 0.006 and 0.027, respectively) in pT1 patients, whereas we did not observe any survival benefit conferred by second TUR in pTa patients. Table 3 summarizes uni- and multivariate models predicting RFS, PFS, CSS and OS in the pT1 cohort. Older age (HR 1.02, 95% CI 1.01–1.03, *P* = 0.005), recurrent tumour (HR 1.70, 95% CI 1.26–2.29, *P* < 0.001), multiple tumours (HR 1.49, 95% CI 1.18–1.88, *P* < 0.001) and larger tumour (HR 1.52, 95% CI 1.21–1.92, *P* < 0.001) were significant adverse factors for intravesical recurrence. Maintenance BCG (HR 0.47, 95% CI 0.33–0.66, *P* < 0.0001) and second TUR (HR 0.80, 95% CI 0.64–1.00, *P* = 0.048) significantly reduced intravesical recurrence in the multivariate model. Regarding PFS, older age (HR 1.03, 95% CI 1.01–1.05, *P* < 0.001) and recurrent tumour (HR 1.46, 95% CI 1.00–2.11, *P* = 0.048) were significant risk factors for disease progression, and maintenance BCG significantly reduced disease progression on multivariate analysis (HR 0.56, 95% CI 0.35–0.90, *P* = 0.016). Although the patients with second TUR had a lower risk of disease progression on univariate analysis (HR 0.75, 95% CI 0.56–1.00, *P* = 0.049), it lost significance in the multivariate model (HR 0.82, 95% CI 0.61–1.10, *P* = 0.18). In terms of CSS, older age (HR 1.08, 95% CI 1.05–1.11, *P* < 0.0001) and recurrent tumour (HR 1.92, 95% CI 1.16–3.18, *P* = 0.011) were independently significant risk factors for cancer-specific mortality. Although patients with second TUR had a lower risk of cancer death on univariate analysis (HR 0.54, 95% CI 0.35–0.85, *P* = 0.007), it became a marginal value in the multivariate model (HR 0.64, 95% CI 0.41–1.01, *P* = 0.057). An older age (HR 1.08, 95% CI 1.06–1.10, *P* < 0.0001) and a recurrent tumour (HR 1.71, 95% CI 1.21–2.41, *P* = 0.002) were significant adverse risk factors for OS.

**Table 3:** Univariate and multivariate Cox proportional hazards model predicting intravesical recurrence, disease progression, cancer-specific mortality and overall mortality in patients with pT1 disease

	*N*	**Intravesical recurrence**				**Disease progression**				**Cancer-specific mortality**				**Overall mortality**			
		Univariate		Multivariate		Univariate		Multivariate		Univariate		Multivariate		Univariate		Multivariate	
		HR (95% CI)	*P-*value	HR (95% CI)	*P-*value	HR (95% CI)	*P-*value	HR (95% CI)	*P-*value	HR (95% CI)	*P-*value	HR (95% CI)	*P-*value	HR (95% CI)	*P-*value	HR (95% CI)	*P-*value
Age (years) (Each increase of 1 year)		1.02 (1.01–1.03)	**<0.0001**	1.02 (1.01–1.03)	**0.005**	1.03 (1.02–1.05)	**<0.001**	1.03 (1.01–1.05)	**<0.001**	1.08 (1.05–1.11)	**<0.0001**	1.08 (1.05–1.11)	**<0.0001**	1.08 (1.06–1.10)	**<0.0001**	1.08 (1.06–1.10)	**<0.0001**
Sex Male Female	1220 267	Ref. 0.95 (0.74–1.22)	0.69			Ref. 0.91 (0.62–1.33)	0.61			Ref. 0.86 (0.48–1.52)	0.60			Ref. 0.67 (0.45–1.01)	0.053		
Tumour status Primary Recurrent	1282 205	Ref. 1.98 (1.57–2.49)	**<0.0001**	Ref. 1.70 (1.26–2.29)	**<0.001**	Ref. 1.58 (1.10–2.27)	**0.014**	Ref. 1.46 (1.00–2.11)	**0.048**	Ref. 2.27 (1.38–3.72)	**0.001**	Ref. 1.92 (1.16–3.18)	**0.011**	Ref. 1.90 (1.36–2.67)	**<0.001**	Ref. 1.71 (1.21–2.41)	**0.002**
Pathological tumour grade Low High	33 1453	Ref. 0.99 (0.51–1.91)	0.97			Ref. 1.99 (0.49–8.01)	0.33			Ref. 1.43 (0.20–10.28)	0.72			Ref. 1.13 (0.36–3.54)	0.83		
Concurrent CIS No Yes	1015 465	Ref. 1.09 (0.89–1.34)	0.38			Ref. 1.21 (0.90–1.63)	0.22			Ref. 0.92 (0.57–1.48)	0.73			Ref. 1.02 (0.75–1.38)	0.90		
Tumour multiplicity Single Multiple	509 931	Ref. 1.48 (1.20–1.83)	**<0.001**	Ref. 1.49 (1.18–1.88)	**<0.001**	Ref. 1.32 (0.96–1.81)	0.086			Ref. 1.43 (0.89–2.32)	0.14			Ref. 1.17 (0.87–1.59)	0.30		
Tumour size <3 cm ≥3 cm	907 339	Ref. 1.32 (1.05–1.65)	**0.017**	Ref. 1.52 (1.21–1.92)	**<0.001**	Ref. 1.20 (0.85–1.70)	0.31			Ref. 1.29 (0.78–2.13)	0.32			Ref. 1.05 (0.74–1.48)	0.80		
Maintenance BCG No Yes	1247 240	Ref. 0.47 (0.34–0.64)	**<0.0001**	Ref. 0.47 (0.33–0.66)	**<0.0001**	Ref. 0.53 (0.33–0.84)	**0.008**	Ref. 0.56 (0.35–0.90)	**0.016**	Ref. 0.85 (0.46–1.57)	0.61			Ref. 0.78 (0.51–1.17)	0.23		
No. BCG instillations at induction ≥6 <6	1377 105	Ref. 1.19 (0.83–1.70)	0.35			Ref. 0.78 (0.41–1.47)	0.44			Ref. 1.35 (0.62–2.93)	0.44			Ref. 1.29 (0.78–2.16)	0.32		
BCG strain used Tokyo–172 Connaught	1133 349	Ref. 1.10 (0.89–1.36)	0.37			Ref. 0.95 (0.69–1.31)	0.77			Ref. 0.76 (0.46–1.23)	0.26			Ref. 0.93 (0.68–1.25)	0.61		
Second TUR No Yes	650 837	Ref. 0.73 (0.60–0.88)	**0.001**	Ref. 0.80 (0.64–1.00)	**0.048**	Ref. 0.75 (0.56–1.00)	**0.049**	Ref. 0.82 (0.61–1.10)	0.18	Ref. 0.54 (0.35–0.85)	**0.007**	Ref. 0.64 (0.41–1.01)	0.057	Ref. 0.73 (0.55–0.97)	**0.027**	Ref. 0.85 (0.64–1.14)	0.27

The *p*-values with significant differences were listed in bold.

**Figure 2:**
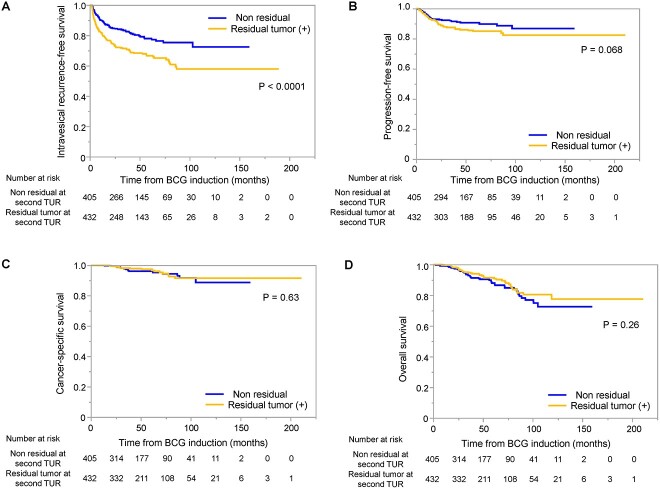
Kaplan–Meier survival analysis displaying (A) intravesical RFS, (B) PFS (C) CSS and (D) OS in T1 bladder cancer patients with or without residual tumours at second transurethral resection.

Figure 2 shows the survival impact of a residual tumour at the second TUR in pT1 patients. A residual tumour at the second TUR was associated with a higher intravesical recurrence rate.

## Discussion

It is well-known that several major clinical guidelines strongly recommend second TUR for high-risk NMIBC patients. In the present cohort, 56.3% (837/1487) of pT1 bladder cancer patients received second TUR, whereas 16.7% (166/995) of pTa bladder cancer patients received second TUR, indicating the gap between evidence-based decision-making and real-world clinical practice. As described before, residual tumours were detected in 53.6% of pTa patients (89/166), and 51% of pT1 patients (430/837) being in line with previous observations of 17–67% residual tumour detection rates (7,10–13). Whilst, in the entire cohort, it did not remain a significant prognostic factor with improved RFS, PFS and CSS in the multivariate model, second TUR independently reduced intravesical recurrence rates in pT1 patients. CSS was also improved in the pT1 cohort with second TUR, although the *P*-value was marginal in the multivariate model (*P* = 0.057).

Recently, Eroglu et al. (14) reported the long-term survival outcomes of a randomized controlled trial that investigated the role of re-TUR in pT1 bladder cancer at a 10-year follow-up. The 10-year OS rate was significantly higher in patients undergoing second TUR (59.1 vs. 40.8%, respectively, *P* = 0.004), and second TUR (OR 1.66, 95% CI 1.16–2.39, *P* = 0.006) remained an independent factor of prolonged OS in the multivariate model. In their study, mitomycin C instillation was utilized for disease prevention. In a Canadian population-based retrospective observational cohort study (*n* = 7666), Wettstein et al. (15) also reported that OS and CSS rates were significantly higher in patients who underwent re-TUR during any time of follow-up. Even after adjusting for background variables, re-TUR was significantly correlated with lower overall mortality (HR 0.88, 95% CI 0.81–0.95, *P* < 0.001), whereas the details of intravesical treatment were not described in their paper. Therefore, to our knowledge, our study involves one of the largest series evaluating the survival impact of second TUR in conjunction with BCG treatment.

On the other hand, several researchers do not recommend a routine second TUR procedure. For example, Gontero et al., in their pT1 HG bladder cancer patients treated with BCG (*n* = 2451), observed that second TUR conferred a survival advantage only in patients without the presence of muscularis propria in the first TURBT specimen. Yanagisawa et al. (16) also recently reported that second TUR after en bloc resection of bladder tumour for pT1 bladder cancer could not improve either recurrence or progression. Taken together with their observations, we should keep in mind that high-quality TUR, which means it includes the muscularis propria for accurate disease staging and disease eradication by meticulous procedures, is mandatory before bladder preservation treatment. Although our group believes in the significant role of second TUR, their observations are important, and the indication of second TUR may be discussed based on initial TUR methods, quality, age or comorbidity in actual clinical practice. Needless to say, TUR is not a risk-free procedure, with a 5% rate of postoperative complications, such as hematuria, infection, bladder injury or urinary retention (17).

In line with previous studies (18,19), maintenance BCG significantly reduced intravesical recurrence and disease progression both in the entire and pT1 cohorts. Because of the low compliance with maintenance therapy, several researchers assessed reduced dose or reduced frequency regimens. For example, the results of the NIMBUS trial were recently published (20). NIMBUS was the first prospective study including a routine repeat TUR in the protocol, and 91% of patients underwent second TUR before BCG instillation. The NIMBUS study assessed whether a reduced frequency of BCG instillations (induction at 1, 2 and 6 weeks followed by 2 weeks of maintenance at 3, 6 and 12 months) revealed equivalent outcomes to the standard frequency (6 weekly instillations followed by 3 weeks of maintenance at 3, 6 and 12 months) in HG NMIBC patients. Disease recurrence was noted in 21 of 175 patients in the standard frequency cohort, and 46 of 170 in the reduced frequency cohort, and safety analyses revealed that the reduced frequency regimen was inferior to the standard schedule for the primary end-point according to the previously used stop criterion. In the present study, as we did not have details of the maintenance schedule and complete rate of planned regimens, we could not draw a definitive conclusion regarding the ideal maintenance schedule. What we can at least say is that ‘adequate BCG instillation’ should always be considered to maximize its clinical benefit.

Regarding the survival impact of residual tumours at a second TUR, we observed that a residual tumour at second TUR was associated with a higher intravesical recurrence rate (*P* < 0.0001), and higher disease progression rate with a marginal *P*-value (*P* = 0.068) in pT1 patients, in line with our previous study (single institution) (7). Several researchers also reported similar results whereby the pathology on second TUR provides prognostic information (21–23). Theoretically, a tumour-free status is achieved in most cases. In some proportion, residual disease at second TUR may reflect very rapid recurrence with a highly aggressive nature. We consider that patients with residual cancer at second TUR require close follow-up, and larger studies are necessary to confirm its survival impact.

Our study had several limitations, including the retrospective nature of analysis, lack of central pathology review, heterogeneity of skill level amongst surgeons and detailed surgical records were not collected. We did not have strict prospective rules for tumour size estimation. In addition, second TUR was performed based on each physicians’ discretion, the details of the time intervals between the initial and second TUR were unknown, performance of maintenance BCG was not standardized and details of maintenance BCG treatment (planned schedule, completion rate, etc.) were not collected. Nevertheless, this study is one of the largest series demonstrating the survival benefit of second TUR in conjunction with BCG treatment.

## Conclusions

Second TUR confers accurate pathological staging and could be used to safely select good candidates for intravesical BCG instillation. We further confirm that second TUR could confer an oncological benefit in pT1 bladder cancer patients treated by BCG instillation, and so strongly recommend second TUR in this patient population.

## Supplementary Material

supplementary_materials_hyad155

## Data Availability

The data analyzed during the present study are not publicly available for the protection of personal data.
