# Supplementary figures and images for: The impact of second transurethral resection on survival outcomes in patients with non-muscle-invasive bladder cancer treated with bacillus Calmette–Guérin therapy
Source: Jpn J Clin Oncol. 2023 Nov 16;54(2):192–200. doi: 10.1093/jjco/hyad155 (PMC10849170; doi:10.1093/jjco/hyad155)

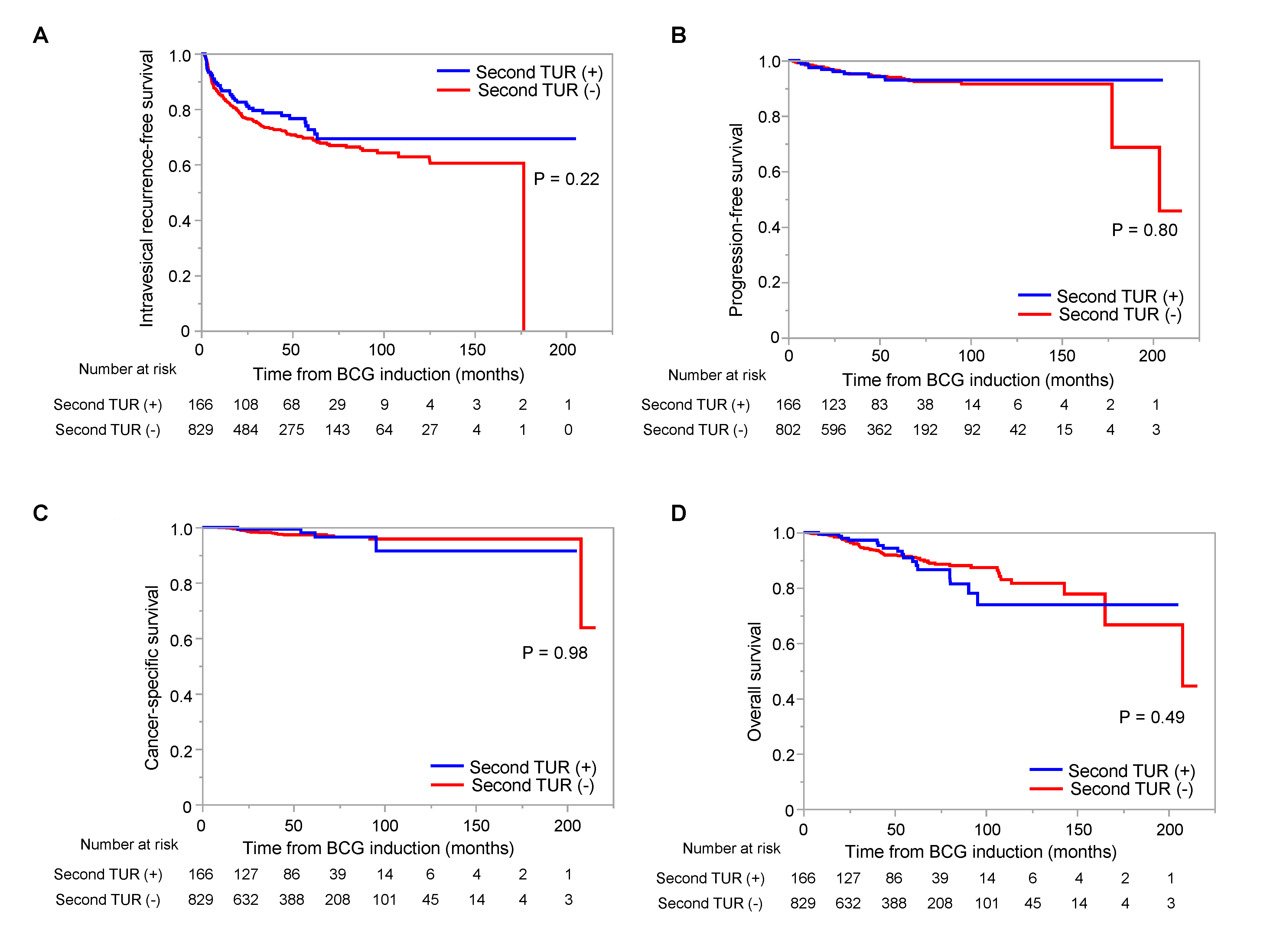

Supplement: supplementary_materials_hyad155 [file supplementary_materials_hyad155.zip › supplementary_materials_hyad155/Supplementary Figure_1.tif]
