# Supplementary material for: The impact of second transurethral resection on survival outcomes in patients with non-muscle-invasive bladder cancer treated with bacillus Calmette–Guérin therapy
Source: Jpn J Clin Oncol. 2023 Nov 16;54(2):192–200. doi: 10.1093/jjco/hyad155 (PMC10849170; doi:10.1093/jjco/hyad155)
Supplement: supplementary_materials_hyad155 [file supplementary_materials_hyad155.zip › supplementary_materials_hyad155/Supplementary Table 1.doc]

**Supplementary Table 1. Pathological results of second TUR**

|  | **T stage at first TUR** | | |
| --- | --- | --- | --- |
|  | Ta (n=166) | T1 (n=837) | CIS (n=23) |
| **T stage at second TUR, n (%)** |  |  |  |
| No residual | 77 (46.4) | 405 (48.4) | 6 (26.1) |
| CIS | 24 (14.5) | 136 (16.2) | 9 (39.1) |
| pTa | 58 (34.9) | 145 (17.3) | 4 (17.4) |
| pT1 | 7 (4.2) | 149 (17.8) | 4 (17.4) |
| Unknown | 0 (0.0) | 2 (0.2) | 0 (0.0) |
